# Spatial Structure of Evolutionary Models of Dialects in Contact

**DOI:** 10.1371/journal.pone.0134335

**Published:** 2015-07-29

**Authors:** Yugo Murawaki

**Affiliations:** Department of Advanced Information Technology, Graduate School of Information Science and Electrical Engineering, Kyushu University, Fukuoka, Japan; Hellas, GREECE

## Abstract

Phylogenetic models, originally developed to demonstrate evolutionary biology, have been applied to a wide range of cultural data including natural language lexicons, manuscripts, folktales, material cultures, and religions. A fundamental question regarding the application of phylogenetic inference is whether trees are an appropriate approximation of cultural evolutionary history. Their validity in cultural applications has been scrutinized, particularly with respect to the lexicons of dialects in contact. Phylogenetic models organize evolutionary data into a series of branching events through time. However, branching events are typically not included in dialectological studies to interpret the distributions of lexical terms. Instead, dialectologists have offered spatial interpretations to represent lexical data. For example, new lexical items that emerge in a politico-cultural center are likely to spread to peripheries, but not vice versa. To explore the question of the tree model’s validity, we present a simple simulation model in which dialects form a spatial network and share lexical items through contact rather than through common ancestors. We input several network topologies to the model to generate synthetic data. We then analyze the synthesized data using conventional phylogenetic techniques. We found that a group of dialects can be considered tree-like even if it has not evolved in a temporally tree-like manner but has a temporally invariant, spatially tree-like structure. In addition, the simulation experiments appear to reproduce unnatural results observed in reconstructed trees for real data. These results motivate further investigation into the spatial structure of the evolutionary history of dialect lexicons as well as other cultural characteristics.

## Introduction

The similarity between the evolution of biological species and that of natural languages has long been noted. Both Charles Darwin’s “Origin of Species” [1] and August Schleicher’s Indo-European family trees [2] were published in the middle of the 19th century. Successful computational models to infer evolutionary history have been developed for biological data [3] and then applied to languages. Such models can also be extended to other cultural elements including manuscripts [4], folktales [5], material cultures [6], and religions [7]. With respect to language applications, phylogenetic models have been used to study the evolutionary histories of Indo-European [8, 9], Austronesian [10], and Bantu languages [11, 12], among others.

In phylogeny, trees are the standard model. The tree model is characterized by a series of branching events in which two or more copies of a node are created and subsequently evolve in separate processes. Traits are passed from a common ancestor to its descendants as attribute changes occur over time. In other words, the tree model explains the shared traits of descendants by depicting common ancestors.

A fundamental question about the tree model is whether it is a suitable approximation of evolutionary history. In particular, linguists have long questioned such models. Shortly after the publication of Schleicher’s language trees, Johannes Schmidt proposed the rivaling *wave theory* [13]. According to the wave theory, linguistic changes spread like waves from a center of origin to its neighbors. This indicates that shared linguistic traits are a result of contact. Despite the rise of computational phylogenetic models, linguists still consider the wave theory as a necessary complement to the tree model [14, 15].

Several attempts have been made to model this contact as an additional layer over the tree, where contact-induced changes are referred to as horizontal transmission. It is well known in biology that horizontal gene transfer plays an important role in the evolution of prokaryotes and it can also occur in eukaryotes [16]. An analogy in linguistics is the effort to explain the idiosyncratic position of Germanic languages within the Indo-European language-family tree with regard to contact with distantly-related Italo-Celtic languages [17].

Even though most phylogenetic models do not take horizontal transmission into account, several simulation studies have asserted their robustness with respect to horizontal transmission [18–21]. When horizontal transmission is added to a reference tree, phylogenetic models can reconstruct the original tree with some degree of accuracy. Inferring a generalized graph with horizontal transfer instead of using a tree remains a challenging task, despite some attempts [22, 23]. Thus, a suitable compromise involves supplementing the reference tree with edges representing horizontal transfer. This approach was originally proposed for prokaryote evolutionary models but has also been applied to languages [24].

The computational models explained above assume tree-like evolution overall, even if horizontal transmission is included. The validity of this fundamental assumption for linguistic applications has been questioned. For example, while the tree model may be applicable to large language families such as Indo-European and Austronesian in which linguistic divergences coincide with population expansion [9, 10], its applicability to groups of dialects or language varieties that maintain high degrees of mutual intelligibility is dubious. It has been noted that the tree model is insufficient for modeling dialects in contact [22, 25, 26]. Still, some publications show application of the tree model to particular groups of dialects including Japonic [27] and Ainu [28]. Although [29] point out that the reconstructed Japonic tree is incompatible with other linguistic evidence, its validity has not been analyzed from a computational point of view.

**Fig 1:**
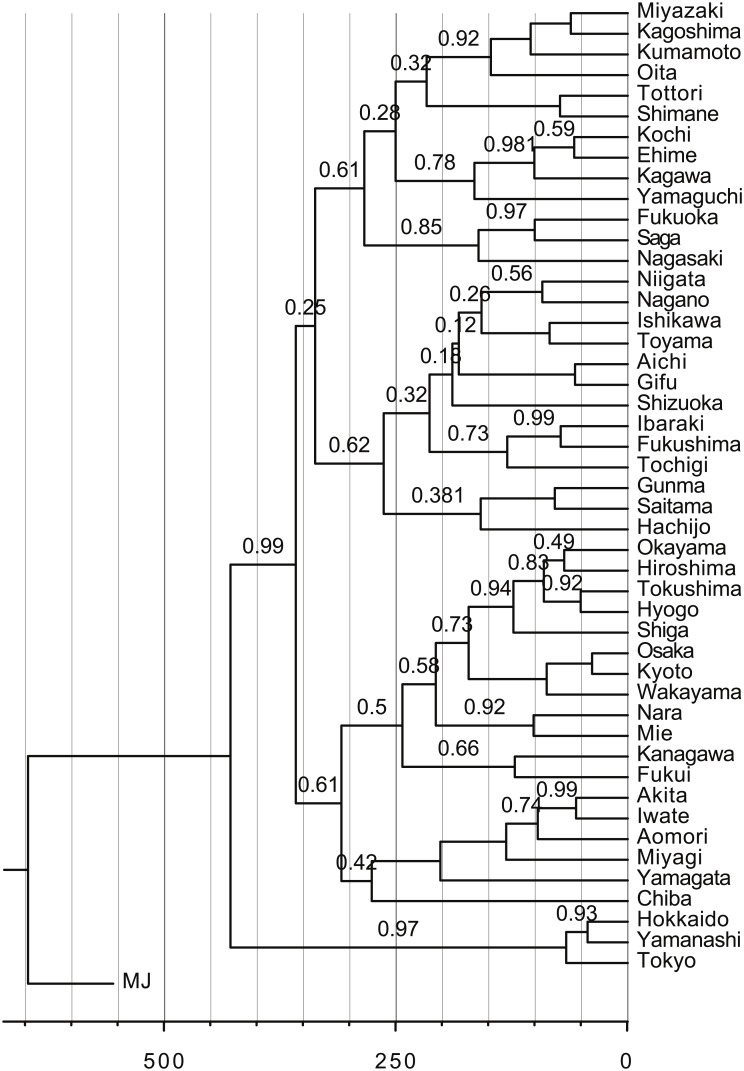
Maximum clade credibility tree of Japonic dialects. The horizontal axis gives the time before present. The number attached to each branch is the posterior sample support ratio for the corresponding clade. The data were taken from Table s3 of Lee and Hasegawa [27]. We tried to replicate the original experiments, but there are minor differences between Lee and Hasegawa’s and ours probably because of the random nature of the sampling process and unexplained model configurations. These variances do not affect the overall results or our discussion.

To highlight the obstacles to applying tree models to dialects in contact, we examine a reconstructed tree for Japonic dialects. We exclude Ryukyuan dialects and show the mainland portion of the tree in Fig 1 (hereafter the mainland portion of Japonic is simply referred to as Japanese). Among several drawbacks of the resulting tree, it is particularly inconsistent that modern Japanese dialects are interpreted as having been formed by a series of branching events in the past 500 years. These branching events are unlikely to be explained by historical events like mass migrations. If these purported branching events do not correspond to key events in historic times, it seems too optimistic to expect that they correspond in prehistoric times. However, the date of the first branching event was associated with the arrival of the first farmers to the Japanese archipelago [27]. It is almost certain that all of the data points considered (except the northernmost point of Hokkaido) had Japanese speakers in the past 500 years without massive population flows. In this example, it is clear that it is more consistent to keep all of the data points intact in the recent past than to merge them into common ancestors.

If a branching structure does not adequately model the formation of modern dialects, how should linguistic evolution be modeled? We believe that the answer lies in dialectology—dialect geography in particular. Dialectologists have investigated the geographic distributions of lexical and grammatical features [30, 31]. In fact, the main portion of Japonic lexical data used in phylogenetic analysis comes from this line of research [32]. While dialectologists occasionally offer speculations about historical changes, the field study data are a current snapshot of distributions, since earlier states are not directly observable. Still, dialectology favors *spatial* interpretations over branching events to explain the progression to current distributions. For example, if a lexical term is observed at multiple isolated points, it is considered to have had a wider geographic distribution in the past [33], which is reminiscent of the wave theory.

Several dialectology studies attempting to track changes in traits among dialect groups have been published. In these studies, a phenomenon known as *dialect leveling* has been investigated extensively [34, 35]. Dialect leveling refers to the reduction of differences between dialects in contact. Studies on dialect leveling have two significant limitations. First, the time scale of the data is limited to the order of decades. Since most dialects have not been documented adequately, dialectologists are forced to track the dynamics themselves. Second, identifying borrowed terms between closely related dialects is generally difficult or impossible, with the exception of certain individual lexical items. We conjecture that this is why the main research focus has been on phonological variations rather than lexicons. For these reasons, our research uses inferences from the field study snapshots rather than tracked changes.

We present a simple simulation model inspired by dialectological studies to explore the tree model’s validity. The model we propose is a drastic departure from the tree model in that branching events are essentially absent from this model. Instead, dialects form a spatial network in which they share lexical terms through contact, rather than through common ancestors. The evolutionary dynamics are characterized by network topologies: some dialects are more influential than others and some pairs of dialects are more distant than others. The network topologies allow us to generate snapshots of lexical distributions. We then input this synthetic data to phylogenetic models to analyze how they interpret the data. While these simulation experiments are not aimed at definitively refuting the tree model as a viable structure for linguistic evolution, we do intend to demonstrate that exploring spatial structure is a promising research direction.

## Materials and Methods

### Basic Vocabulary

In a phylogenetic model, each language is represented by a set of traits. We model languages with concepts constituting the basic vocabulary [36, 37]. Words representing each basic concept are assumed to be relatively stable. A basic vocabulary database is compiled by recording the words that represent each basic concept in each target language. Next, cognates (words which have a common origin) are determined for the group of target languages. For example, in Japanese dialects, the concept “warm” is represented by “attage” in Aomori, “atatakai” in Tokyo, “nukui” in Osaka and “nukka” in Fukuoka. The words for “warm” in Aomori and Tokyo are cognates. The same is true for “warm” in Osaka and Fukuoka. The words deemed cognates are denoted by a 1 and a 0 is assigned where no cognate is found. This allows each language to be transformed into a binary vector such as 10010…. The distance between a pair of languages is defined as the ratio of digits that are different between the two vectors. Such binary vectors form the input to the phylogenetic models. A language may have multiple cognates for a single concept.

A disadvantage of this representation is that it ignores the fact that certain sets of cognates bear the same concept. In the examples above, the concept “warm” is represented by two cognates, but this can no longer be recovered from the binary vectors. To overcome this, we use concept-based modeling to attach weights to cognates signifying the same concepts.

### Simulation Model

We propose a simple simulation to model changes in dialect lexicons resulting from contact. While a continuous-time framework is dominant in phylogeny, we adopt a discrete-time model for simplicity. This model forms a Markov chain: the state at time *t* only depends on the state at time *t* − 1.

**Fig 2:**
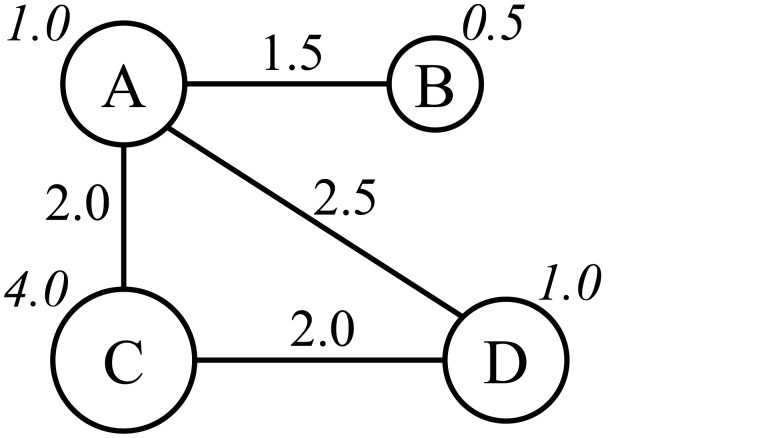
Undirected graph of nodes.

We consider a network of nodes, each of which represents a dialect. Fig 2 shows an example of network topologies. Each node’s state is a set of cognates that represents a given concept. Let $V_{i}^{t}\neq \emptyset$ be the set of cognates for node *i* at time *t* ≥ 0. Here we assume that each dialect has at least one cognate for the given concept. Each node behaves like an agent and stochastically decides its next state depending on the neighboring nodes. Nodes in contact are connected by edges, as illustrated in Fig 2. The network topology is time-invariant in most cases, but it can be changed over time, as we will see below. Time-invariant topologies are the default choice since we are unable to synthesize realistic data for historical population dynamics.

**Fig 3:**
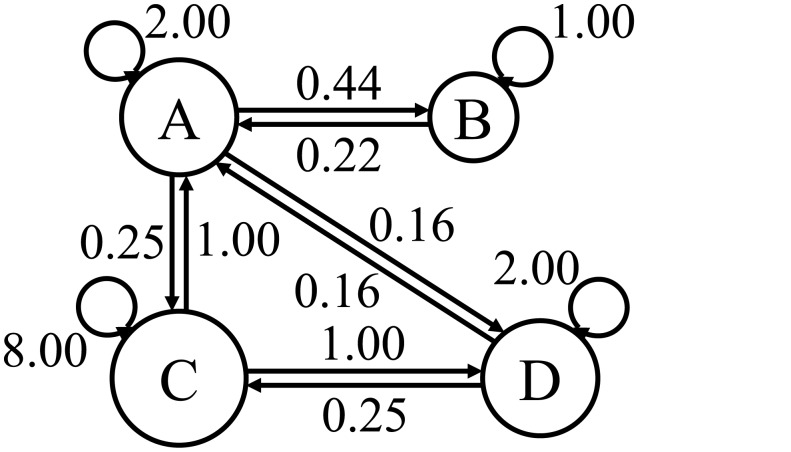
Directed graph of nodes. We set *s* = 2.0.

The degree of each node’s influence is controlled by two parameters: population and distance. In Fig 2, the numbers attached to nodes and edges denote populations and distances, respectively. Let *p*_*i*_ be node *i*’s population, and *d*_*ij*_ = *d*_*ji*_ be the distance between *i* and *j*. The influence of node *j* on node *i* can be defined as

$$w_{ij}=\{\begin{array}{cc} p_{j}\;/\;\left(d_{ij}\times d_{ij}\right) & \text{if}j\in N_{i},\\ s\;p_{i} & \text{if}j=i,\\ 0 & \text{otherwise}, \end{array}$$

where *N*_*i*_ is the set of nodes connected to node *i* (node *i* is not included in the set). A larger population leads to a greater influence on neighbors and influence decays with distance. Parameter *s* controls the influence on the node itself. The directed graph in Fig 3 shows the degree of influence *w*_*ij*_.

The state of node *i* at time *t* > 0 stochastically depends on the state of the network at time *t* − 1. The network state at time *t* = 0 is given a priori. For simplicity, we assume that all nodes share the same single cognate at time *t* = 0.

Let $C_{i}^{t}$ be a set of candidate cognates within node *i* at time *t*. The candidates of $V_{i}^{t}$ are the power set of $C_{i}^{t}$ excluding the empty set:

$$V_{i}^{t}\in 𝓟\left(C_{i}^{t}\right)\backslash \emptyset .$$

$C_{i}^{t}$ is constructed by combining a new cognate candidate with sets of cognates that node *i*’s neighbors had at time *t* − 1.

$$C_{i}^{t}=\{\text{newword}\}\cup \underset{j\in \{i\}\cup N_{i}}{⋃}V_{j}^{t-1}.$$

Here we assume that at most one new cognate appears in a node at each time step. A newly introduced cognate (birth) is used only in a single node. Once a cognate disappears from all nodes (death), it can never revive.

To prepare for a formal definition of the probability of the state of $v\in 𝓟(C_{i}^{t})\backslash \emptyset$, we give the following score to each cognate $c\in C_{i}^{t}$:

$$\text{wscore}\left(c\right)=\{\begin{array}{cc} \sum_{j\in M_{i,c}^{t}}w_{ij}/\left|V_{j}^{t-1}\right| & \text{if }c\neq \text{newword},\\ b\;p_{i} & \text{otherwise}, \end{array}$$

where $M_{i,c}^{t}=\left\{j\;|\;j\in \{i\}\cup N_{i}\;\wedge \;c\in V_{j}^{t-1}\right\}$. In other words, $M_{i,c}^{t}$ is the set of neighboring nodes, including *i*, that has *c* at time *t* − 1. The score is directly proportional to the number of nodes containing *c* at time *t* − 1. The score of a new cognate is controlled by parameter *b*.

Next, we assign a score to the set of cognates $V_{i}^{t}=v$:

$$\text{sscore}\left(v\right)=\sum_{c\in v}(\text{wscore}\left(c\right)-d^{\left|v\right|}).$$

Cost *d* > 1 is used to penalize a set with a larger number of cognates. Finally, the probability of choosing $V_{i}^{t}=v$ is defined as:

$$\frac{\exp(\text{sscore}(v))}{\sum_{v^{\prime }\in 𝓟\left(C_{i}^{t}\right)\backslash \emptyset }\exp\left(\text{sscore}\left(v^{\prime }\right)\right)}.$$

$V_{i}^{t}$ is chosen according to the distribution above. At each time step, this procedure is performed for all nodes in the network.

The number of steps *T* is pre-defined. In our experiments, we set *T* = 1,000. When the simulation terminates, we obtain the distributions of cognates for one concept. We repeat this for all of the basic concepts in our list and form binary representations of the resulting cognate distributions. We thus obtain binary representations of the dialects. Following the Swadesh list [36, 37], we use 100 basic concepts.

This model relies on several assumptions that might be violated in real data. It assumes that for each concept, every dialect has one or more words that precisely refer to it. However, some dialects may have no generic term for the concept (e.g., using different words when the concept is used in reference to humans or animals). These words complement each other rather than competing as the model assumes. Borrowings from external languages can usually be interpreted as births. If the same cognate is borrowed multiple times, it violates the assumption that a cognate comes into existence only once. New cognates do not necessarily emerge spontaneously and may result from semantic shifts of existing words. Such semantic shifts can happen multiple times. Despite these possible violations of the model assumptions, we contend that they are exceptions.

The proposed model is closely related to word-of-mouth communication, in which information is passed over a network. While we model words themselves, previous studies have worked on marketing and public opinion formation [38–40]. For this reason, existing publications focused mainly on maximizing the spread of influence. Thus, their simulation models include some assumptions that are not suitable for modeling the spread of words. For example, most of them only model the birth and spread of a trait, ignoring its death. By contrast, we consider the death of a trait as an important aspect of linguistic evolutionary dynamics. In addition, they often focus on a single trait while we model a competition among several traits.

Another closely related research area is evolutionary game theory. In particular, our model resembles an evolutionary graph, or evolutionary game involving spatial structure [41]. This field focuses on modeling the strategy chosen by an individual from a population at some point of time. Parameters given a priori include a number (typically two) of strategies and the corresponding payoff matrix. Evolutionary graphs also have a network topology as a given parameter. The research focus is on finding a small number of parameters that characterize the overall evolutionary dynamics (e.g., the probability of a trait spreading over the entire population). Words in our model can be seen as game strategies. It should be noted that, because of the emergence of new words in our model, the number of strategies cannot be given a priori.

### Network Topologies

We employ five network topologies in our simulation: Grid, Star, TwoStars, Bottleneck and Colony. Hereafter, undirected graphs are used to show populations and distances.

**Fig 4:**
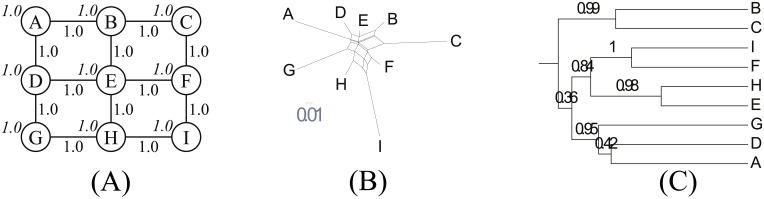
Grid. (A) Network topology. We set *s* = 4.0, *b* = 1.0 and *d* = 3.0. (B) NeighborNet. (C) Reconstructed tree.

Grid (Fig 4A) is a grid of nodes with the equivalent population sizes. It is an example of a spatially non-tree-like structure.

**Fig 5:**
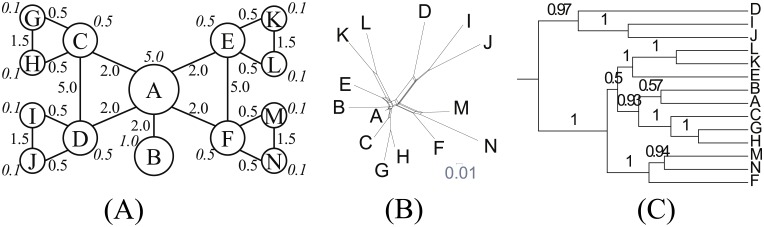
Star. (A) Network topology. We set *s* = 2.0, *b* = 0.1 and *d* = 3.0. (B) NeighborNet. (C) Reconstructed tree.

In Star (Fig 5A), a node with a large population is surrounded by several small nodes. They represent a politico-cultural center and peripheries, respectively. Unlike Grid, this topology is spatially tree-like. Note that there is no bottleneck in the topology that can be interpreted as a branching event. This challenges the phylogenetic model’s requirement to construct a temporal tree.

**Fig 6:**
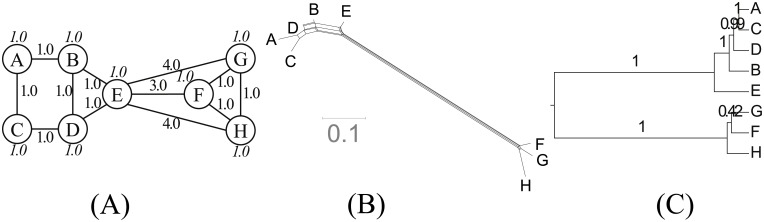
Bottleneck. (A) Network topology. We set *s* = 4.0, *b* = 0.1 and *d* = 3.0. (B) NeighborNet. (C) Reconstructed tree.

Bottleneck (Fig 6A) contains a bottleneck between two groups of dialects. Unlike Star, this can be interpreted by a phylogenetic model as a branching event that occurs at the beginning of the simulation. The key is that the two groups are not totally disconnected and keep in contact on a small scale. We are interested in how this affects phylogenetic analysis.

**Fig 7:**
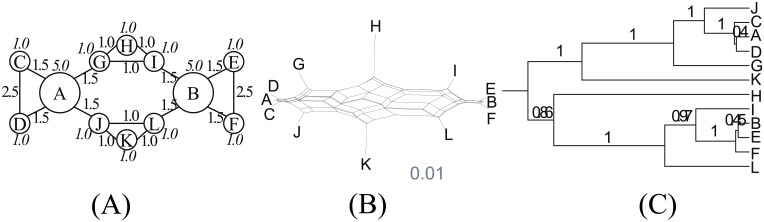
TwoStars. (A) Network topology. We set *s* = 2.0, *b* = 1.0 and *d* = 3.0. (B) NeighborNet. (C) Reconstructed tree.

TwoStars (Fig 7A) is a variant of Star. Two centers, not just one, are presented to see how the nodes in between are interpreted.

**Fig 8:**
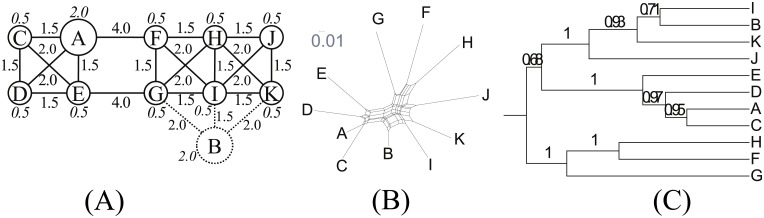
Colony. (A) Network topology. We set *s* = 4.0, *b* = 0.1 and *d* = 3.0. (B) NeighborNet. (C) Reconstructed tree.

Colony (Fig 8A) undergoes a change in the network topology. The first 750 steps out of 1,000 steps are performed without node B (indicated by dashed lines). Then node B is copied from node A, and the remaining 250 steps are performed. The replication can be interpreted as a branching event by a phylogenetic model. Note that this network topology consists of two dialect groups. Node B can be seen as a large colony of the western dialect group in the east. Our focus is on how this affects phylogenetic inference.

### Analytical Methods

We use phylogenetic methods to analyze the binary representations of dialects obtained through our simulations. Following [26], we first assess the degree to which the data follow a tree-like structure using the *δ* score [42]. We thus obtain a real number between 0 and 1 inclusive. Lower values indicate that the data are more tree-like. The basis of the *δ* score is a quartet of nodes *x*, *y*, *u* and *v*. Let *d*_*xy*_ be the distance between *x* and *y*, and *d*_*xy*∣*uv*_ = *d*_*xy*_ + *d*_*uv*_. Without loss of generality, we assume *d*_*xy*∣*uv*_ ≤ *d*_*xu*∣*yv*_ ≤ *d*_*xv*∣*yu*_ and define *δ*_*xyuv*_ as follows:

$$\delta_{xyuv}=\frac{d_{xv|yu}-d_{xu|yv}}{d_{xv|yu}-d_{xy|uv}}.$$

**Fig 9:**
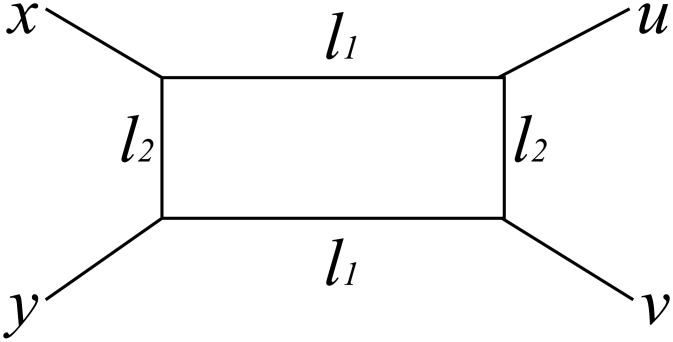
A diagram in which the distances between four nodes are decomposed.

Note that we set *δ*_*xyuv*_ = 0 if *d*_*xy*∣*uv*_ = *d*_*xu*∣*yv*_ = *d*_*xv*∣*yu*_. To illustrate the above, the distances between nodes can be decomposed as shown in Fig 9. The box indicates non-tree-likeness. Here *l*_1_ = *d*_*xv*∣*yu*_ − *d*_*xy*∣*uv*_, *l*_2_ = *d*_*xv*∣*yu*_ − *d*_*xu*∣*yv*_ and *δ*_*xyuv*_ = *l*_2_/*l*_1_. The quartet is perfectly tree-like if *l*_2_ = 0. *δ*_*xyuv*_ is calculated for every possible quartet and is averaged to obtain the overall *δ* score.

Unfortunately, there is no known statistical test that effectively compares two or more *δ* scores [26]. We thus compare the *δ* scores of our synthetic data with those reported in existing publications: 0.25 for Ainu dialects [28], 0.22 for Indo-European languages as a whole and 0.23 for Germanic languages, and 0.41 for the Polynesian branch of Austronesian languages [26]. We calculate the *δ* scores for the Japonic dialects using supplementary information since they are not reported [27]. We obtained 0.35 for the whole dataset and 0.39 for the modern mainland dialects.

Next, we use NeighborNet [43] to visualize the relationship between dialects. NeighborNet performs distance-based agglomerative clustering and constructs a network instead of a tree. Non-tree-likeness is indicated by boxes. SplitsTree4 version 4.13.1 was used [44] for the implementation.

Finally, we apply a Bayesian phylogenetic model to the data. It is a generative tree model that directly models evolutionary processes. For inference, samples are obtained from a posterior distribution. We use maximum clade credibility trees to integrate multiple samples for visualization. We use a stochastic Dollo model, in which the birth of a trait (a cognate in our case) happens only once in the tree. This means that once a node loses a cognate, it will not be recovered by a descendant. In the simulation model, however, a cognate that has disappeared from a node can be revived through contact with neighboring nodes. The prior distribution for the death rate of the Dollo model is an exponential distribution with a mean of 10^−5^. We use a Bayesian skyline prior and a strict clock model. Markov chain Monte Carlo sampling is performed for 20 million steps. After the burn-in of the first 100,000 steps we collect a sample every 1,000 steps. We use BEAST 1.8.0 to implement the above [45].

## Results

As we expected, Grid is not tree-like. Its *δ* score is 0.43. Its deviation from a tree-like structure is confirmed in NeighborNet (Fig 4B). Node E, the center of the network topology is also centrally located in the recovered structure. The four corners, which have less contact with neighbors, are distantly positioned. The non-tree-likeness is also reflected in the reconstructed tree (Fig 4C) as clades near the root are unstable.

Star is moderately tree-like with a *δ* score of 0.28. NeighborNet (Fig 5B) recovers the spatial structure. Node A, the center of the network topology is also centrally located in NeighborNet. These results suggest that tree-likeness can result from a spatial tree structure, rather than just temporal relationships. The reconstructed tree (Fig 5C) reinterprets this spatial structure as a temporal structure. Spatially distant nodes are shown as having split at earlier stages.

Bottleneck is very tree-like. Its *δ* score is 0.15. NeighborNet (Fig 6B) recovers two dialect groups. Among the group of nodes A–E, node E is closest to the group containing F, G and H. The two dialect groups are also recovered in the phylogenetic tree (Fig 6C) but here node E is interpreted as an outlier of the dialect group. The clustering of the node with an intermediate state was delayed by the Bayesian phylogenetic model, just like simple agglomerative clustering. We conjecture that a similar effect can explain the reconstructed phylogenetic tree for Ainu dialects [28]. In this tree, Soya (Hokkaido’s northernmost point and the nearest point to Sakhalin) is treated as Hokkaido’s outlier. Our simulation study suggests that this could result from a spatial structure rather than a temporal structure. This implication challenges Lee and Hasegawa’s conclusion that northern Hokkaido was the most likely homeland based on a joint inference of the geolocations of ancestral nodes. Soya, which is supposed to have split from the Hokkaido group at the earliest time, might have contributed to the northward move of the estimated geolocations of Proto-Hokkaido and Proto-Ainu.

TwoStars is tree-like with a *δ* score of 0.19. NeighborNet (Fig 7B) does not appear tree-like at first glance but the lower score is indicated by the long and thin boxes in the network. The phylogenetic tree (Fig 7C) reconstructs two dialect groups. Note the locations of nodes K and H. Although they are equidistant from the two centers in the network topology, they are clustered into different groups. They are both outliers in their corresponding groups yet the reconstructed tree is quite stable. This suggests that random effects have a considerable impact on the overall result.

Colony has a *δ* score of 0.33, and is not tree-like. Nodes A and B, which split at a later stage, are not distant in NeighborNet (Fig 8B). However, the reconstructed tree (Fig 8C) splits them at a very early stage. Many posterior samples separate A and B at the earliest split, as indicated by a relatively low support score of 0.68 for the I, B, K, J, E, D, A, and C clades. Thus, the interpretation presented by the tree is that most nodes in the network were formed after the split of A and B, even though they existed from the very beginning. This is a possible explanation for the reconstructed tree of mainland Japanese dialects in Fig 1. The earliest split, except for pre-modern languages, separates Tokyo from Kyoto despite the model’s assumption that they split relatively recently [27]. At the time of the split, node B represented the western dialect group and subsequently dominated the eastern lexicon of the neighboring nodes. This effectively blurred the distinction between the two dialect groups, as demonstrated by the unstable reconstructed tree. We conjecture the same occurrences for mainland Japanese dialects since Fig 1 partially fails to identify the distinction between Eastern and Western Japanese [46].

## Discussion

The key findings of our simulation experiments are 2-fold. First, low *δ* scores do not necessarily indicate a temporally tree-like evolution. In fact, spatially tree-like structures account for the low *δ* scores. Second, some network topologies lead to apparently unnatural results observed in the reconstructed trees of the real data. These results are sufficient to cast doubt on the applicability of the temporal tree model to dialects. Still, we concede that more accurate evolutionary scenarios than ours may exist. This motivates a further investigation of spatial structure in the evolutionary history of dialect lexicons as well as other cultural traits.

Our future research will focus on two improvements. First, we have used the model only for simulation purposes, but the model parameters should be inferred from real data. To make such inferences tractable, we may require a more simplified model. Model parameters include population, distance, and the number of steps. Agreement between these parameters and the real data is still uncertain while phylogenetic models successfully employ time calibration and, recently, inferences about geolocations. Second, we aim to undertake the challenge of definitively determining the model that best fits the real data: the temporal tree, the spatial network, or a hybrid model.

In addition to these main directions of research, the incorporation of linguistic hypotheses deserves further study. For example, *prestige* is often cited as a key factor leading to borrowings [14]. Such hypotheses should be tested with simulation experiments.

Another potential improvement involves replacing the node representing a dialect with a network of individuals. In the current model, each node represents a dialect which is used by multiple speakers. The asymmetry among dialects is controlled solely by the population parameter. This model design is based on descriptive linguistics, in which a language is chronicled for a population rather than for individuals. This marks a sharp contrast with biology, where one population can be represented by multiple DNA samples. Genetic variations within a population provide important information for evolutionary history [47, 48]. We hypothesize that the per-individual approach is not a preferred choice for linguistics for two reasons. First, we cannot expect a high degree of linguistic variation within a population since language is a communication medium. Clearly, speakers need to share lexical items for successful communication. Second, describing a language is laborious and requires linguistic experts, and is therefore costly. Given that real data are difficult to obtain, simulation experiments are a suitable alternative.

**Fig 10:**
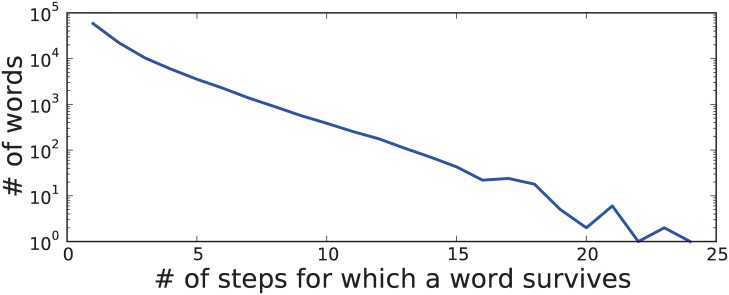
Survival times for words in Star. Survival time is defined as the number of steps between birth and death. The words used in the initial and final states were excluded.

There are several matters to address in the current simulation model. Fig 10 depicts the distribution of survival times in Star. We see that a large number of short-lived words are required to allow some words to survive over long periods of time. This is because new words are often quickly replaced by existing words surviving in neighboring nodes. In the current simulation model, new words are less likely to enter nodes with larger populations. Since the probability distribution is constructed using an exponential function, larger nodes have more skewed distributions. New words generally have smaller scores and thus have much lower probabilities. However, once new words are accepted in a large node, they are likely to spread to surrounding nodes. On the one hand, this phenomenon seems to be supported by the astonishingly diversified dialects of Ryukyuan, which have been spoken by a hundreds of small, isolated populations [49]. On the other hand, despite its small population, Icelandic is known as a very conservative language [50]. Thus, more evidence is required to support or refute our assumptions.

In Fig 1, we excluded Ryukyuan dialects from consideration since our constant contact assumption may not be valid for the dialects of small, remote islands. Still, it is clear that contact-induced changes cannot be neglected in studying the evolution of Ryukyuan. The split of Ryukyuan from mainland Japanese is believed to predate Old Japanese (the language of central Japan in the 8th Century) [51, 52]. This theory is based on a traditional comparative approach focusing on regular sound correspondences. However, Ryukyuan dialects share a considerable number of lexical items with mainland Japanese, including some grammaticalized words, even though these terms postdate Old Japanese [53–55]. Thus, they were likely borrowed from mainland Japanese dialects. Contact-based models like ours could aid in unraveling the mysterious evolution of Ryukyuan dialects.

## References

[1] Darwin C. The Origin of Species by Means of Natural Selection or, the Preservation of Favored Races in the Struggle for Life. John Murray; 1859.

[2] Schleicher A. Die ersten Spaltungen des indogermanischen Urvolkes. Allgemeine Monatsschrift für Wissenschaft und Literatur. 1853;3:786–787. (in German).

[3] Fitch WM, Margoliash E. Construction of Phylogenetic Trees. Science. 1967;155(760):279–284. 10.1126/science.155.3760.279 5334057

[4] Barbrook AC, Howe CJ, Blake N, Robinson P. The Phylogeny of the Canterbury Tales. Nature. 1998;394:839 10.1038/29667

[5] Ross RM, Greenhill SJ, Atkinson QD. Population Structure and Cultural Geography of a Folktale in Europe. Proceedings of the Royal Society B: Biological Sciences. 2013;280 (1756). 10.1098/rspb.2012.3065 23390109 PMC3574383

[6] Tëmkin I, Eldredge N. Phylogenetics and material cultural evolution. Current Anthropology. 2007;48(1):146–154. 10.1086/510463

[7] Matthews LJ. The Recognition Signal Hypothesis for the Adaptive Evolution of Religion. Human Nature: An Interdisciplinary Biosocial Perspective. 2012;23(2):218–249. 10.1007/s12110-012-9138-8 22623139

[8] Gray RD, Atkinson QD. Language-tree Divergence Times Support the Anatolian Theory of Indo-European Origin. Nature. 2003;426(6965):435–439. 10.1038/nature02029 14647380

[9] Bouckaert R, Lemey P, Dunn M, Greenhill SJ, Alekseyenko AV, Drummond AJ, et al Mapping the Origins and Expansion of the Indo-European Language Family. Science. 2012;337(6097):957–960. 10.1126/science.1219669 22923579 PMC4112997

[10] Gray RD, Jordan FM. Language Trees Support the Express-train Sequence of Austronesian Expansion. Nature. 2000;405(6790):1052–1055. 10.1038/35016575 10890445

[11] Holden CJ. Bantu Language Trees Reflect the Spread of Farming across sub-Saharan Africa: a Maximum-Parsimony Analysis. Proceedings of the Royal Society of London Series B: Biological Sciences. 2002;269(1493):793–799. 10.1098/rspb.2002.1955 11958710 PMC1690959

[12] Currie TE, Meade A, Guillon M, Mace R. Cultural Phylogeography of the Bantu Languages of Sub-Saharan Africa. Proceedings of the Royal Society B: Biological Sciences. 2013;280 (1762). 10.1098/rspb.2013.0695 PMC3673054 23658203

[13] Schmidt J. Die Verwandtschaftsverhältnisse der indogermanischen Sprachen. Hermann Böhlau; 1872. (in German).

[14] Campbell L. Historical Linguistics: An Introduction (2nd edition). Edinburgh University Pres; 2004.

[15] Labov W. Transmission and Diffusion. Language. 2007;83(2):344–387. 10.1353/lan.2007.0082

[16] Andersson JO. Lateral Gene Transfer in Eukaryotes. Cellular and Molecular Life Sciences CMLS. 2005;62(11):1182–1197. 10.1007/s00018-005-4539-z 15761667 PMC11138376

[17] Ringe D, Warnow T, Taylor A. Indo-European and Computational Cladistics. Transactions of the Philological Society. 2002;100(1):59–129. 10.1111/1467-968X.00091

[18] Nicholls GK, Gray RD. Dated Ancestral Trees from Binary Trait Data and their Application to the Diversification of Languages. Journal of the Royal Statistical Society: Series B (Statistical Methodology). 2008;70(3):545–566. 10.1111/j.1467-9868.2007.00648.x

[19] Greenhill SJ, Currie TE, Gray RD. Does Horizontal Transmission Invalidate Cultural Phylogenies? Proceedings of the Royal Society B: Biological Sciences. 2009;276(1665):2299–2306. 10.1098/rspb.2008.1944 19324763 PMC2677599

[20] Currie TE, Greenhill SJ, Mace R. Is Horizontal Transmission Really a Problem for Phylogenetic Comparative Methods? A Simulation Study Using Continuous Cultural Traits. Philosophical Transactions of the Royal Society B: Biological Sciences. 2010;365(1559):3903–3912. 10.1098/rstb.2010.0014 PMC2981909 21041214

[21] Barbançon F, Warnow T, Evans SN, Ringe D, Nakhleh L. An Experimental Study Comparing Linguistic Phylogenetic Reconstruction Methods. Diachronica. 2013;30(2):143–170. 10.1075/dia.30.2.01bar

[22] Nakhleh L, Ringe D, Warnow T. Perfect Phylogenetic Networks: A New Methodology for Reconstructing the Evolutionary History of Natural Languages. Language. 2005;p. 382–420. 10.1353/lan.2005.0078

[23] Warnow T, Evans SN, Ringe D, Nakhleh L. A Stochastic Model of Language Evolution that Incorporates Homoplasy and Borrowing In: Forster P, Renfrew C, editors. Phylogenetic Methods and the Prehistory of Languages. McDonald Institute for Archaeological Research; 2006 p. 75–90.

[24] Nelson-Sathi S, List JM, Geisler H, Fangerau H, Gray RD, Martin W, et al Networks Uncover Hidden Lexical Borrowing in Indo-European Language Evolution. Proceedings of the Royal Society B: Biological Sciences. 2010;. 10.1098/rspb.2010.1917 21106583 PMC3097823

[25] Nichols J, Warnow T. Tutorial on Computational Linguistic Phylogeny. Language and Linguistics Compass. 2008;2(5):760–820. 10.1111/j.1749-818X.2008.00082.x

[26] Gray RD, Bryant D, Greenhill SJ. On the Shape and Fabric of Human History. Philosophical Transactions of the Royal Society B: Biological Sciences. 2010;365(1559):3923–3933. 10.1098/rstb.2010.0162 PMC2981918 21041216

[27] Lee S, Hasegawa T. Bayesian Phylogenetic Analysis Supports an Agricultural Origin of Japonic Languages. Proceedings of the Royal Society B: Biological Sciences. 2011;278(1725):3662–3669. 10.1098/rspb.2011.0518 21543358 PMC3203502

[28] Lee S, Hasegawa T. Evolution of the Ainu Language in Space and Time. PLoS ONE. 2013;8(4):e62243 10.1371/journal.pone.0062243 23638014 PMC3637396

[29] Whitman J. Northeast Asian Linguistic Ecology and the Advent of Rice Agriculture in Korea and Japan. Rice. 2011;4(3–4):149–158. 10.1007/s12284-011-9080-0

[30] Trudgill P. The Dialects of England, 2nd edition Blackwell Publishers; 2000.

[31] Upton C, Widdowson JDA. An Atlas of English Dialects: Region and Dialect. Routledge; 2006.

[32] Hirayama T, editor. Gendai Nihongo hōgen daijiten. Meiji Shoin; 1992.

[33] Yanagita K. Kagyu-kō. Tōkō Shoin; 1930.

[34] Kerswill P. Dialect levelling and geographical diffusion in British English In: Britain D, Cheshire J, editors. Social dialectology: in honour of Peter Trudgill. John Benjamins Publishing; 2003 p. 223–243.

[35] Labov W. Transmission and Diffusion. Language. 2007;83(2):344–387. 10.1353/lan.2007.0082

[36] Swadesh M. Lexicostatistic Dating of Prehistoric Ethnic Contacts. Proceedings of American Philosophical Society. 1952;96:452–463.

[37] Swadesh M. The Origin and Diversification of Language. Aldine Atherton; 1971.

[38] Kempe D, Kleinberg J, Éva Tardos. Maximizing the Spread of Influence Through a Social Network. In: Proceedings of the Ninth ACM SIGKDD International Conference on Knowledge Discovery and Data Mining; 2003. p. 137–146.

[39] Watts DJ, Dodds PS. Influentials, Networks, and Public Opinion Formation. Journal of Consumer Research. 2007;34(4):441–458. 10.1086/518527

[40] Delre SA, Jager W, Bijmolt THA, Janssen MA. Will It Spread or Not? The Effects of Social Influences and Network Topology on Innovation Diffusion. Journal of Product Innovation Management. 2010;27(2):267–282. 10.1111/j.1540-5885.2010.00714.x

[41] Nowak MA, Tarnita CE, Antal T. Evolutionary Dynamics in Structured Populations. Philosophical Transactions of the Royal Society B: Biological Sciences. 2010;365(1537):19–30. 10.1098/rstb.2009.0215 PMC2842709 20008382

[42] Holland BR, Huber KT, Dress A, Moulton V. *δ* Plots: A Tool for Analyzing Phylogenetic Distance Data. Molecular Biology and Evolution. 2002;19(12):2051–2059. 10.1093/oxfordjournals.molbev.a004030 12446797

[43] Bryant D, Moulton V. Neighbor-Net: An Agglomerative Method for the Construction of Phylogenetic Networks. Molecular Biology and Evolution. 2004;21(2):255–265. 10.1093/molbev/msh018 14660700

[44] Huson DH, Bryant D. Application of Phylogenetic Networks in Evolutionary Studies. Molecular Biology and Evolution. 2006;23(2):254–267. 10.1093/molbev/msj030 16221896

[45] Drummond AJ, Rambaut A. BEAST: Bayesian Evolutionary Analysis by Sampling Trees. BMC Evolutionary Biology. 2007;7(1):214 10.1186/1471-2148-7-214 17996036 PMC2247476

[46] Tojo M. Kokugo no hōgen kukaku. Tōkyōdō Shuppan; 1927.

[47] Wright S. Isolation by Distance. Genetics. 1943;28(2):114 17247074 10.1093/genetics/28.2.114 PMC1209196

[48] Excoffier L, Smouse PE, Quattro JM. Analysis of Molecular Variance Inferred from Metric Distances among DNA Haplotypes: Application to Human Mitochondrial DNA Restriction Data. Genetics. 1992;131(2):479–491. 1644282 10.1093/genetics/131.2.479 PMC1205020

[49] Nakamoto M. Zusetsu Ryūkyūgo jiten. Kinkeisha; 1981.

[50] Karlsson S. The Icelandic Language. Viking Society for Northern Research; 2004.

[51] Serafim LA. The Use of Ryukyuan in Understanding Japanese Language History In: Frellesvig B, Whitman J, editors. Proto-Japanese: Issues and Prospects. John Benjamins Publishing; 2008 p. 79–99.

[52] Shimabukuro M. The Accentual History of the Japanese and Ryukyuan Languages: A Reconstruction. Global Oriental; 2007.

[53] Kamei T. Ryūkyū hōgen no shiteki chii In: Nihongo keitōron no michi. Yoshikawa-Kōbunkan; 1973 p. 91–114.

[54] Nakamoto M. Ryūkyūgo no seiritsu to jidaisō: Nihongo no genzō o saguru In: Ryūkyū goi-shi no kenkyū. San-ichi Publishing; 1983 p. 15–27.

[55] Ogino C. A Study of Yari-Morai Constructions in the Honorific-Priority System, especially Focusing on Taborun in the Yaeyama Ryukyuan and on Tamawaru in Pre-Modern Japanese. Studies in the Japanese language. 2011;7(4):39–54.

